# Analysis of the convective heat transfer through straight fin by using the Riemann-Liouville type fractional derivative: Probed by machine learning

**DOI:** 10.1016/j.heliyon.2024.e25853

**Published:** 2024-02-08

**Authors:** Waseem, Asad Ullah, Sabir Ali, Fuad A. Awwad, Emad A.A. Ismail

**Affiliations:** a School of Mechanical Engineering, Jiangsu University, Zhenjiang 212013, Jiangsu, China; b School of Finance and Economics, Jiangsu University, 301, Xuefu Road, Jingkou District, Zhenjiang 212013, Jiangsu, China; c Department of Mathematical Sciences, University of Lakki Marwat, Lakki Marwat, 28420, Khyber Pakhtunkhwa, Pakistan; d National University of Modern Languages, Islamabad, Pakistan; e Department of Quantitative Analysis, College of Business Administration, King Saud University, P.O. Box 71115, Riyadh 11587, Saudi Arabia

**Keywords:** Heat transfer, Convection, Straight fin, Porous surface, Artificial intelligence, Fractional differential equations, Artificial neural network

## Abstract

This work aims to analyze the transfer of heat through new fractional-order convective straight fins by using the Riemann-Liouville type fractional derivatives. The convection through the fins is considered in such a way that the thermal conductivity depends on the temperature. The transformed fractional-order problems are constituted through an optimization problem in such a way that the $L_{2}$ norm remains minimal. The objective functions are further analyzed with the hybrid Cuckoo search (HCS) algorithm that use the artificial neural network (ANN) mechanism. The impacts of the fractional parameter *β*, the thermo-geometric parameter of fin *ψ*, and dimensionless thermal conductivity *α* are explained through figures and tables. The fin efficiency during the whole process is explained with larger values of *ψ*. It is found that the larger values of *ψ* decline the fin efficacy. The fractional parameter declines the thermal profile as we approach the integer order. The convergence of HCS algorithm is performed in each case study. The residual error touches E−14 for the integer order of *α*. The present results are validated through Table 6 by comparing with HPM, VIM and LHPM, while the error for HCS-ANN touches E−13. This proves that the proposed HCS is efficient.

## 1 Introduction

Non-linear differential equations are used to simulate most scientific situations, such as heat transport [1]. Heat transfer is an essential and helpful subject in mechanical engineering since it is necessary for many things. The rate of convective heat transfer may be increased using several techniques for example, with the help of heat transfer coefficient or by increasing the cross-sectional area. It is well accepted that the surface area for heat transfer may be increased by attaching fins made of highly conductive materials to the ground sheet. Fins are built in such a way that they improve heat transmission from the ground sheet to the surroundings. Besides playing a key role in the heat transfer rates, fins also provide efficacy in the rejection of heat in many systems and can help cool many types of electronic instruments and space spacecraft [2], [3]. Kern and Kraus [4] published a full study in this regard in a monograph. Domairry and Fazeli [5] attempted to investigate the effectiveness of straight fin convection by using HPM. Chiu and Chen [6] investigated convective longitudinal fins with varying thermal conductivity using the Adomian's decomposition technique (ADM). In another work, Chiu and Chen [7] used the decomposition approach to investigate the convective-radiative relationship. The heat-rejecting mechanism was studied by Bartas and Sellers [8]. Furthermore, the radial and straight fins convection is summarized by Coskun and Atay [9] by implementing VIM. Arslanturk [10] examined the best design of space radiators using and demonstrating the efficacy of ADM. Aziz and Hug [11] used the conventional perturbation approach to investigate the efficiency of convective straight fins in another study. In this follow-up, the sumudu transform technique is used by Patra and Ray [12] by using HPM to straight fin efficiency and convection rate.

Several monographs dedicated to derivatives and integrals of fractional orders can be found in the literature [13], [14], [15], [16], [17], [18]. Singh et al. [19] and Carvalho and Pinto [20] proposed a fractional order delay mathematical model for predicting malaria and human immunodeficiency virus co-infection. Srivastava et al. [21] investigated a fractional model vibration equation. Yang et al. [22] used the local fractional derivative (FD) for investigating the KdV equation, and Jafari et al. [23] used the local type fractional operators for the analysis of the differential equations. The relaxation and diffusion type fractional equations are studied by Yang et al. [24]. He et al. [25] discussed the applications of a novel fractional derivative. Later on, this work was used by Wang and Liu [26] for the investigation of fractional heat transfer equations. Further analysis of He's work can be found in the references [27], [28], [29]. A more brief survey of the fractional calculus definitions of various derivatives and integrals can be found in Caputo [30], Yang [31], He [25], [32]. Caputo and Fabrizio [33] have presented a new fractional derivative. The novel derivative is significant since it is required to use a mathematical model to understand the nature of diverse processes. Indeed, the classical Caputo fractional (CF) derivative appears to be particularly appropriate for mechanical phenomena. The physical processes in which these effects are missing appear to be more appropriate for using the innovative definition of fractional derivative. Atangana [34] investigated the nature of Fisher's reaction-diffusion equation using the novel fractional derivative. In another study, Atangana and Koca [35] used this method to the nonlinear Baggs and Freedman model and demonstrated the effectiveness of the novel fractional derivative. Hristov [36], [37] shown in a series of works that the novel Caputo-Fabrizio fractional derivative may be readily produced from the Cattaneo idea of flux relaxation if the damping function is the Jeffrey memory kernel. Sun et al. [38] investigated fractional relaxation and diffusion using non-singular kernels. Yang et al. [39] suggested and tested a novel fractional derivative with a non-singular kernel in constant heat flow. Atangana and Baleanu [40] proposed a novel non-integer order derivative with a non-local and non-singular kernel in another paper. Mirza and Vieru [41] used a mixture of Laplace and Fourier transforms to solve the advection-diffusion equation with a time-fractional Caputo-Fabrizio derivative. Atangana and Baleanu [42] demonstrate the use of a Caputo-Fabrizio derivative in groundwater flow in a limited aquifer. Ali et al. [43] investigated the MHD free convection flow of the generalized Walters'-B fluid model using the Caputo-Fabrizio derivative. Baleanu et al. [44] compared Caputo and Caputo-Fabrizio derivatives for the advection differential equation. In the Allen Cahn model, Algahtani [45] employed the Atangana-Baleanu and Caputo-Fabrizio derivatives with fractional order. Recently, Sing et al. [46] studied the energy balance past a straight fin with LHPM. They modified the HPM by using the Laplace transform and implemented the Caputo-Fabrizio derivatives. They compared their results with VIM and HPM for validation. Brociek et al. [47] used the Riemann–Liouville type fractional derivative to analyze the two dimensional system by considering the alternating direction implicit method (ADIM). They have also identified the unknown parameters by taking the inverse problem. Varun et al. [48] used the internal heat generation through a triangular porous fin for investigating the thermal transport. Abdulrahman et al. [49] studied the longitudinal exponential witted porous fin. Kumar et al. [50], [51] investigated a wavy fin by considering different impacts through a machine learning approach. Rajan et al. [52] used the numerical and optimization strategies to analyze the heat transfer through fin. A more recent survey on the fin analysis can be found in the references [53], [54], [55], [56].

Inspired by the above literature, We present a new fractional model for the energy balance equation associated with the new Riemann-Liouville fractional derivative to calculate the efficiency of convective straight fins with temperature-dependent thermal conductivity. To investigate the energy balance equation of fractional order, the efficiency of ANN coupled with the hybrid Cuckoo search algorithm is used. For different values of various physical parameters, we estimate the non-dimensional temperature distribution and fin tip efficiency for convective straight fins with thermal conductivity. The approach employed is an extension of CS-ANN that uses the hybrid Cuckoo search [57], [58] to handle non-linear PDE associated with novel Riemann-Liouville fractional derivatives. The advantage of the applied approach over traditional analytical techniques is that it takes less computer memory and decreases computing time.

This article is subdivided into section 2 which provides the basic definitions that will be later used in this study. The problem is modeled in section 3, while the ANN mechanism for differential equations and the formulation of the proposed problem into an objective function for minimization of the $L_{2}$ norm is proposed in section 4. The CS algorithm and its hybridization are presented in section 5. The results obtained are presented in section 6, while a conclusion is presented in section 7.

## 2 Basic definitions

There are several definitions of fractional integrals and derivatives in fractional calculus, including Caputo, Riemann Liouville, Hadamard, Riesztype, and Erd-elyi-Kober [17]. Following are some of the famous fractional integrals.

### 2.1 Definition

Suppose that $\theta \in H^{1}(a,b)$, where a>b and β∈[0,1] then, as per Caputo and Fabrizio [33], we have

(1) $$D_{\xi }^{\beta }[\theta (\xi )]=\frac{M(\beta )}{1-\beta }\int_{a}^{\xi }\theta (\tau )e^{\left(-\beta \frac{\xi -\tau }{1-\beta }\right)}d\tau$$

where, M(β) is the normalization function. If $\theta \notin H^{1}(a,b)$, then equation takes the form:

(2) $$D_{\xi }^{\beta }[\theta (\xi )]=\frac{\beta M(\beta )}{1-\beta }\int_{a}^{\xi }(\theta (\xi )-\theta (\tau ))e^{\left(-\beta \frac{\xi -\tau }{1-\beta }\right)}d\tau .$$

The above equation (2) can be re-written for $\gamma =\frac{1-\beta }{\beta }\in [0,\inf)$, $\beta =\frac{1}{1+\gamma }\in [0,1]$ as below [34]:

(3) $$D_{\xi }^{\beta }[\theta (\xi )]=\frac{N(\gamma )}{\gamma }\int_{a}^{\xi }\theta (\tau )e^{\left(\frac{\tau -\xi }{\gamma }\right)}d\tau ,$$

with N(0)=1=N(β) and $\lim_{\gamma ⟶0}(\frac{e^{\frac{-\tau -\xi }{\gamma }}}{\gamma })=\delta (\tau -\xi )$.

The integral discussed in equation (1) is defined by Losada and Nieto [59] and is presented in the following definition.

### 2.2 Definition

The integral operator of order *β*, where β∈(0,1) for a function θ(ξ) can be expressed as [60]:

(4) $$I_{\xi }^{\beta }[\theta (\xi )]=\frac{2}{M(\beta )(2-\beta )}[(1-\beta )\theta (\xi )+\beta \int_{0}^{\xi }\theta (s)ds],\xi \geq 0,$$

and particularly, for $\frac{2(1-\beta )}{\left(2-\beta )M(\beta \right)}+\frac{2\beta }{\left(2-\beta )M(\beta \right)}=1$, we have $M(\beta )=\frac{2}{1-\beta }$ for 0≤β<1. Furthermore, the definition of Caputo-Fabrizio derivative is defined in (1) and can be re-written as:

(5) $$D_{\xi }^{\beta }[\theta (\xi )]=\frac{1}{1-\beta }\int_{a}^{\xi }\theta (\tau )e^{\beta (\frac{\tau -\xi }{1-\beta })}d\tau .$$

The equivalence of these formulations on various functions is proven in [17], [15], [61]. We utilized the Riemann-Liouville formulation of fractional derivatives with a lower terminal at zero in our study. In the literature, the fractional order Riemann-Liouville derivative is defined as:

(6) L[DνRLf(t)]=1Γ(m−ν)dmdtm∫0tf(τ)(t−τ)1+ν−mdτ(m−1<ν≤m),

here, *f* is continuous, ν∈R, n∈N and Γ function is defined as under

(7) $$\Gamma (x)=\int_{0}^{\infty }e^{-t}t^{x-1},R(x)>0.$$

The Mittag-Lifflerfunction (MLF) is significant. It has a wide range of applications in the field of fractional calculus. Its significance is recognized when solving fractional-order differential equations. Podlubny [14] provides the function with two arguments *α* and *β* as:

(8) $$E_{\alpha ,\beta }(t)=\sum_{k=0}^{\infty }\frac{t^{k}}{\Gamma (\alpha k+\beta )},(\alpha >0,\beta >0),$$

and when we take β=1, we have the standard MLF function for one parameter.

## 3 Problems formulation

### 3.1 Problem 1

**Figure 1:**
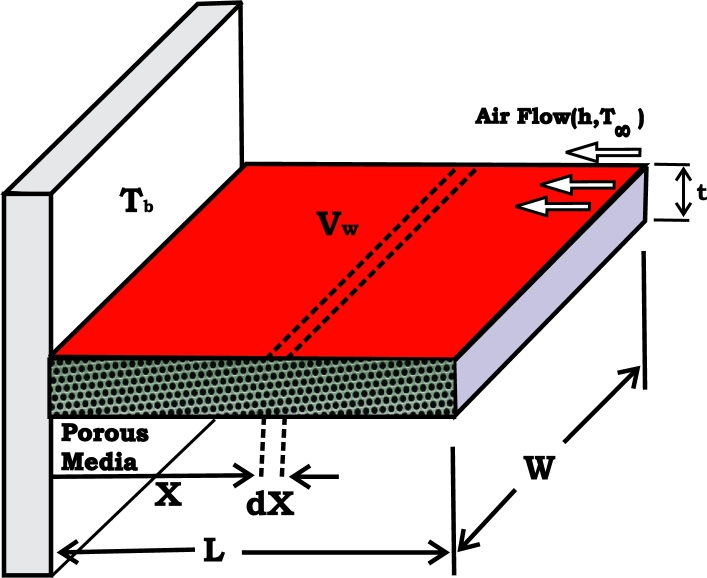
Geometrical description of the problem.

Assume a straight fin with an arbitrary cross-sectional area, $A_{c}$, perimeter *P*, and length *b* is shown in Fig. 1. The fin is connected to the base surface with temperature, $T_{b}$, and extends into fluid with temperature, $T_{a}$, with its insulated tip.

The energy balance equation is thus expressed as follows [9]:

(9) $$\frac{d}{dt}[k(T)\frac{dT}{dx}]-\frac{Ph(T-T_{a})}{A_{c}}=0,$$

here equation (9), k(T) denotes thermal conductivity dependent on temperature, and *h* denotes heat transfer coefficient. It is assumed that the thermal conductivity of the fin material is stated as follows [46]:

(10) $$\frac{k(T)}{k_{b}}-1=\lambda (T-T_{b}).$$

In the relation (10) $k_{b}$ denotes the ambient thermal conductivity, $T_{b}$ is the temperature at a distance from the surface and *λ* is the variational parameter for the thermal conductivity.

Introducing the following dimensionless transformations [44]:

(11) $$\xi =\frac{x}{b},\alpha =\lambda (T_{b}-T_{a}),\theta (T_{b}-T_{a})=(T-T_{a}),\psi ={\left(\frac{Phb^{2}}{k_{a}A_{c}}\right)}^{2}$$

Now, using equation (11) in (9), we have

(12) $$\frac{d^{2}\theta }{d\xi^{2}}+\alpha \theta \frac{d^{2}\theta }{d\xi^{2}}+\alpha {\left(\frac{d\theta }{d\xi }\right)}^{2}-\psi^{2}\theta =0,\;\xi \in [0,1]$$

together with the boundary conditions

(13) $$\frac{d\theta (0)}{d\xi }=0,\;\theta (1)=1.$$

Since, we know that integer order derivatives are local in nature, these derivatives cannot correctly characterize the situation. Because its kernel is non-local and non-singular, the Riemann-Liouville fractional derivative is more suited to describing natural occurrences. As a result, we substitute the second-order derivative in equation (12) with the novel Riemann-Liouville fractional derivative, and this equation (12) changes to a fractional model of energy balance equation written as:

(14) Dξβ+1RL(θ)+αθd2θdξ2+α(dθdξ)2−ψ2θ=0,ξ∈(0,1].

While the boundary conditions remain the same.

#### 3.1.1 Efficiency of the fin

The efficiency of this is a very important physical parameter [46]. To compute its mathematical formula, we use Newton's law of cooling for the rate of heat transfer through the fin. Therefore, we have

(15) $$Q=\int_{0}^{b}P(T-T_{b})dx,$$

The fin efficiency is the ratio of real heat transmitted by the fin to heat transfer if the fin is completely present at room temperature. From above, we have

(16) $$\eta =\frac{Q}{Q_{I}}=\frac{\int_{0}^{b}P(T-T_{b})dx}{(T_{b}-T_{a})Pb}=\int_{0}^{1}\theta (\xi )d\xi .$$

### 3.2 Problem 2

**Figure 2:**
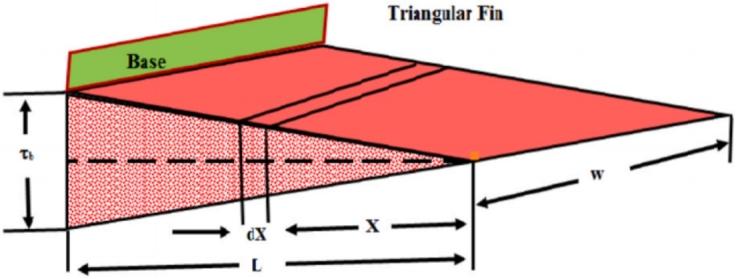
Geometrical description of the problem.

Assume the impacts of convection and radiation through a porous triangular longitudinal fin having cross-section area *A*, length *L* as shown in Fig. 2. Suppose that $T_{s}$, $T_{a}$, *T* and $T_{b}$ are the surface, ambient, fluid and base temperatures, respectively. Furthermore, *k* is the thermal conductivity, *h* is the convective heat transfer coefficient, *ϵ* is the emissivity parameter, *σ* is the Stefan-Boltzmann constant and *ρ* is the density. It is assumed that the fin is moving with a constant velocity *U* in the *x* direction from the base. Under these assumptions, we have [62]:

(17) $$\frac{d^{2}T}{dx^{2}}+\frac{dT}{dx}+x\frac{U}{\lambda }\frac{dT}{dx}-\frac{hP}{Ak_{eff}}\left(T-T_{a}\right)+x\frac{q}{k_{eff}}-\frac{\varepsilon \sigma P}{k_{eff}A}\left(T^{4}-T_{s}^{4}\right)-\frac{\overset{˙}{m}C_{p}}{Ak_{eff}}{\left(T-T_{a}\right)}^{2}=0.$$

The rate of mass flux through the fin is given by

(18) $$\overset{˙}{m}=\rho W△(\frac{\beta Kg}{v}\left(T-T_{a}\right))x.$$

We assume the effective thermal conductivity $k_{eff}=k_{f}\varphi +k_{s}(1-\varphi )$, and the fin temperature differences so small such that

(19) $$\left(T^{2}+T_{s}^{2}\right)\left(T+T_{s}\right)≅T_{b}^{3},$$

Using the above assumptions in equation (17), we have

(20) $$x\frac{d^{2}T}{dx^{2}}+\frac{dT}{dx}+x\frac{U}{\lambda }\frac{dT}{dx}-\frac{hP}{Ak_{eff}}\left(T-T_{a}\right)+x\frac{q}{k_{eff}}-\frac{\varepsilon \sigma PT_{b}^{3}}{k_{eff}A}\left(T-T_{s}\right)-\frac{\rho g\beta C_{p}KW}{vAk_{eff}}{\left(T-T_{a}\right)}^{2}=0.$$

Where, $\lambda =\frac{k_{eff}}{\rho C_{p}}$ is the fin thermal diffusivity.

Assume the following dimensionless group of numbers [62]:

(21) $$\xi =\frac{x}{L},N_{r}=\frac{\varepsilon \sigma PL^{2}T_{b}^{3}}{k_{eff}A},R_{a}=\frac{gd^{3}\beta \left(T-T_{a}\right)}{\lambda v},D_{a}=\frac{k}{d^{2}},S_{p}=\frac{D_{a}{\left(\frac{L}{d}\right)}^{2}R_{a}}{k_{r}},\theta =\frac{T}{T_{b}},\theta_{s}=\frac{T_{s}}{T_{b}},\theta_{a}=\frac{T_{a}}{T_{b}},N^{2}=\frac{hPL^{2}}{Ak_{eff}},Pe=\frac{UPL}{k_{eff}\lambda },$$

here, $C_{p}$ is the specific heat, $\theta_{s},\theta_{a}$ represents the ratios of temperature, *Pe* is the Peclet number, *N* is the conduction-convection, $N_{r}$ is the radiation-conduction and $S_{p}$ is the porosity parameter.

From equation (21), we can write (17) as:

(22) $$\xi \frac{d^{2}\theta }{d\xi^{2}}+(xPe+1)\frac{d\theta }{d\xi }+Qx-N^{2}\left(\theta -\theta_{a}\right)-N_{r}\left(\theta -\theta_{s}\right)-S_{p}{\left(\theta -\theta_{a}\right)}^{2}=0.$$

From above, we have

(23) $$\xi \frac{d^{2}\theta }{d\xi^{2}}+\frac{d\theta }{d\xi }+\xi Pe\frac{d\theta }{d\xi }-\left(N^{2}+N_{r}-2S_{p}\theta_{a}\right)\theta -S_{p}\theta^{2}+N^{2}\theta_{a}+N_{r}\theta_{s}+Q\xi =0.$$

Now introducing the Riemann-Liouville formulation (6), we have

(24) Dξβ+1RL(θ)+1ξdθdξ+Pedθdξ−1ξ(N2+Nr−2Spθa)θ−Spθ2ξ+N2θaξ+Nrθsξ+Q=0.

The B.Cs. corresponding to equation (17) are:

(25) $${T|}_{\xi =L}=T_{b},{\;\frac{dT}{d\xi }|}_{x=0}=1.$$

Implementing equation (21) in above, we have

(26) $$\theta (1)=1,\;\theta^{\prime }(0)=0.$$

## 4 Modeling with ANN and the optimization problem

The solutions to nonlinear problems are well explained in the literature, where various numerical and semi-analytical techniques are explained. The performance of ANN for approximating the solution of nonlinear problems is well explained by Chen et al. [63] and Hornik et al. [64]. This performance for a higher-order data set is explained by Dillon and O'Malley [65], where ANN shows the best approximation.

The main purpose behind the approximation of a solution for a mathematical relation is to find the best approximation that satisfies the given physical system more closely. To achieve this, we need to consider the optimization tools. For this purpose, we introduce a feed-forward algorithm for the fractional order problem (14) as follows [66]:

(27) $$\overset{ˆ}{\theta }(t)=\sum_{i=1}^{m}\;\xi_{i}[f\left(\delta_{i}t+\beta_{i}\right)],$$

(28) $$\frac{d\overset{ˆ}{\theta }(t)}{dt}=\sum_{i=1}^{m}\;\xi_{i}\frac{d}{dt}[f\left(\delta_{i}t+\beta_{i}\right)],$$

(29) $$\frac{d^{2}\overset{ˆ}{\theta }(t)}{dt^{2}}=\sum_{i=1}^{m}\;\xi_{i}\frac{d^{2}}{dt^{2}}[f\left(\delta_{i}t+\beta_{i}\right)],$$

⋮

(30) $$\frac{d^{\beta }\overset{ˆ}{\theta }(t)}{dt^{\beta }}=\sum_{i=1}^{m}\;\xi_{i}\frac{d^{\beta }}{dt^{\beta }}[f\left(\delta_{i}t+\beta_{i}\right)],$$

here, δ,β,ξ are the ANN weights, *m* is the number of neurons, f(t)=exp⁡(−t) is the sigmoid function, and $\overset{ˆ}{\theta }(t)$ is the approximate solution.

Based on the previous analysis, we assume an objective function E to reduce the $L_{2}$ norm for the equations (13)-(14) and (24)-(26). From above, we have

(31) $$Minimize\;E=E_{1}+E_{2},$$

where,

(32) E1=mean[Dξβ+1RL(θˆj)+αθd2θˆjdξ2+α(dθˆjdξ)2−ψ2θˆj]2,

and

(33) $$E_{2}=mean[{\left(\overset{ˆ}{\theta_{1}}-1\right)}^{2}+{\left({\overset{ˆ}{\theta }}_{0}^{\prime }\right)}^{2}].$$

Similarly, for the second problem,

(34) E1=mean[Dξβ+1RL(θˆj)+1ξdθˆjdξ+Pedθˆjdξ−1ξ(N2+Nr−2Spθa)θˆj−Spθˆj2ξ+N2θaξ+Nrθsξ+Q]2,

and $E_{2}$ remains the same as given in equation (33).

**Figure 3:**
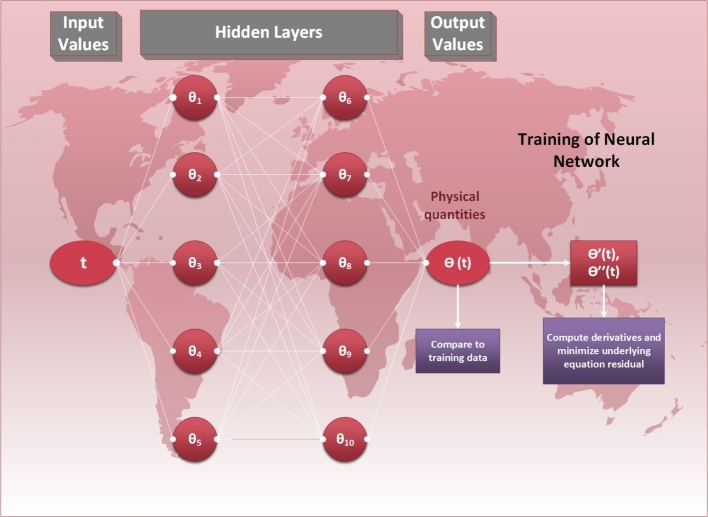
Geometrical description of the ANN for the fractional-order fin temperature model.

Here, $\xi_{j}=jh$, ${\overset{ˆ}{\theta }}_{j}=\overset{ˆ}{\theta }(\xi_{j})$, $N=\frac{1}{h}$ where *h* shows the step size and ξ≥0. The whole mechanism of ANN is described in Fig. 3.

## 5 The Cuckoo search algorithm and its hybridization

As clear from the name Cuckoo, this algorithm uses the Cuckoo search strategy for finding the nest to lay their eggs there. The total search process follows the Lévy flights and random search approach [67]. In some algorithms, like PSO and GA the local optima escapes, and the beauty of this search is that the local optima can not be escaped [68].

This algorithm follows the given search path

(35) $$z_{i}^{t+1}-z_{i}^{t}=\alpha_{1}s⊗H(p_{a}-\epsilon_{1})⊗(z_{j}^{t}-z_{k}^{t}).$$

The Lévy walk equation further enhances the global search for $z_{i}^{t+1}$. Here, H(u) is the Heaviside function, $z_{i}^{t+1}$ and $z_{i}^{t}$ are the two separate roots, $p_{a}$ represents the changing parameter, $\epsilon_{1}$ is the random variable, s>0 is the size of the step taken, ⊗ is the element-wise product, and $\alpha_{1}$ is the scaling parameter.

**Figure 4:**
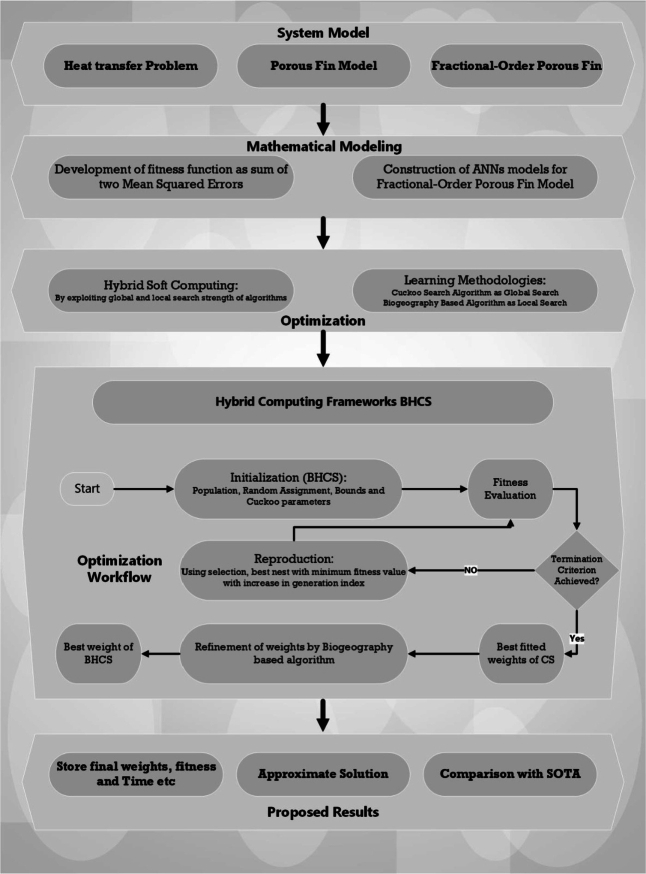
Description of the work to execute the problem efficiently.

The CS algorithm can be further optimized with the Biogeography-based operator. In this methodology, these modified operators are used to build a new optimal solution based on hybrid Cuckoo search (HCS). In the search phase, the host bird is allowed to find the other Cuckoo eggs with higher accuracy to remove the old or to adopt it based on the optimal condition. The total population is again re-evaluated and the rate of emigration *μ* is chosen for each response. This rate can be defined as [69]:

(36) $$P\mu_{i}=\frac{ES_{i}}{M},$$

here, E=1 shows the maximum rate of emigration and Si=MPI is the solution of the species chosen. In the present study, we have fixed the number of nests 30, discovery rate $p_{a}=0.30$, Cuckoo search parameters s=1.1 and $\epsilon_{1}=1.7$ with max number of functions evaluations 120000. The total mechanism of the proposed methodology is explained in Fig. 4.

## 6 Results and discussions

**Figure 5:**
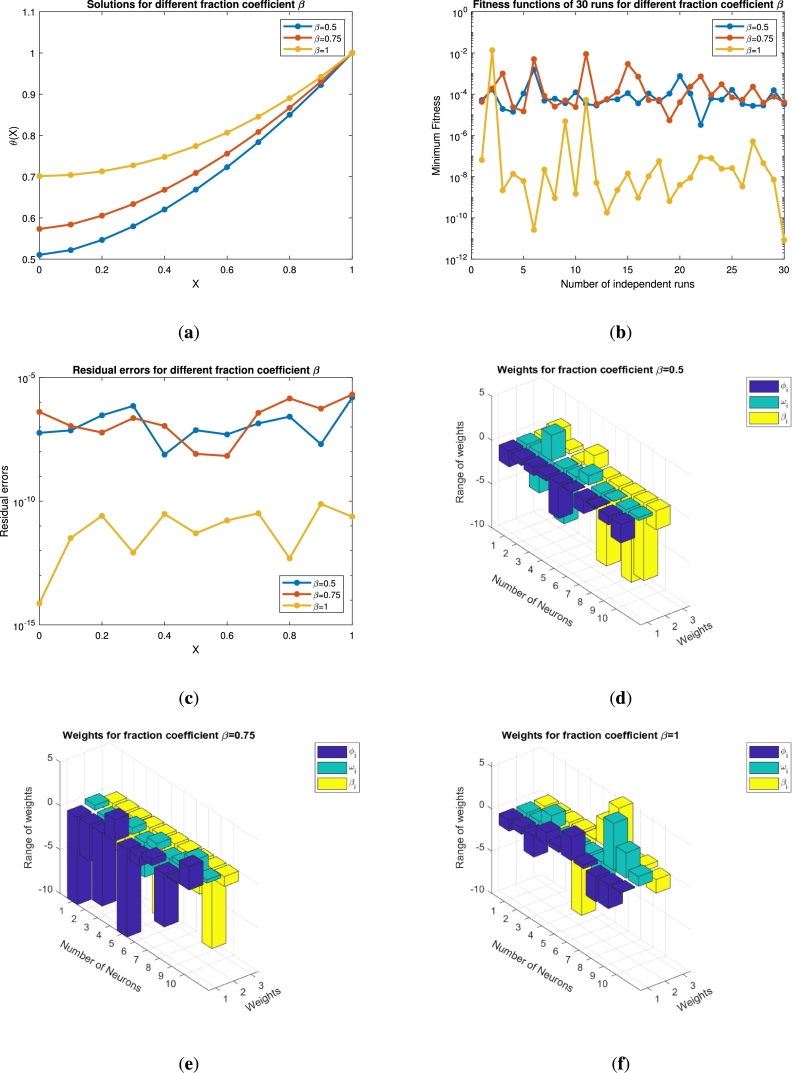
For *β* = 0.5,0.75,1 (**a**) Approximate solution of *θ*(*x*) (**b**) Fitness function (**c**) Error analysis (**d**)-(**f**) Weight functions vs neurons variation.

**Figure 6:**
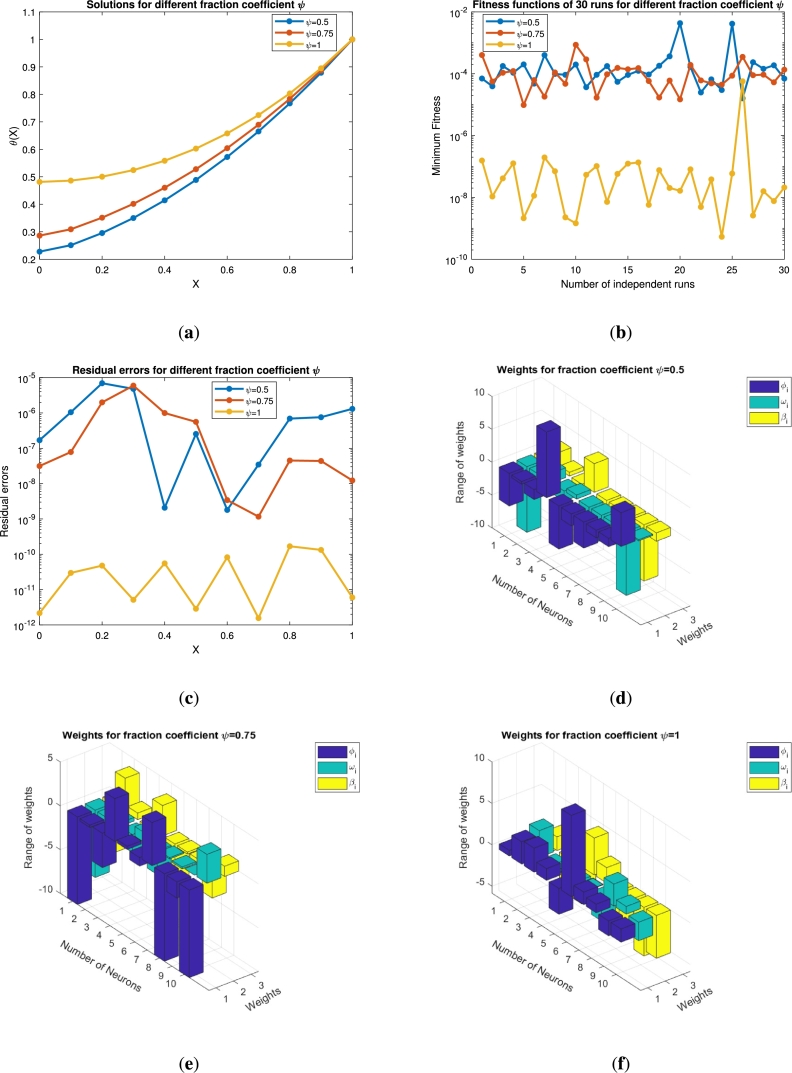
For *ψ* = 0.5,0.75,1 (**a**) Approximate solution of *θ*(*x*) (**b**) Fitness function (**c**) Error analysis (**d**)-(**f**) Weight functions vs neurons variation.

**Figure 7:**
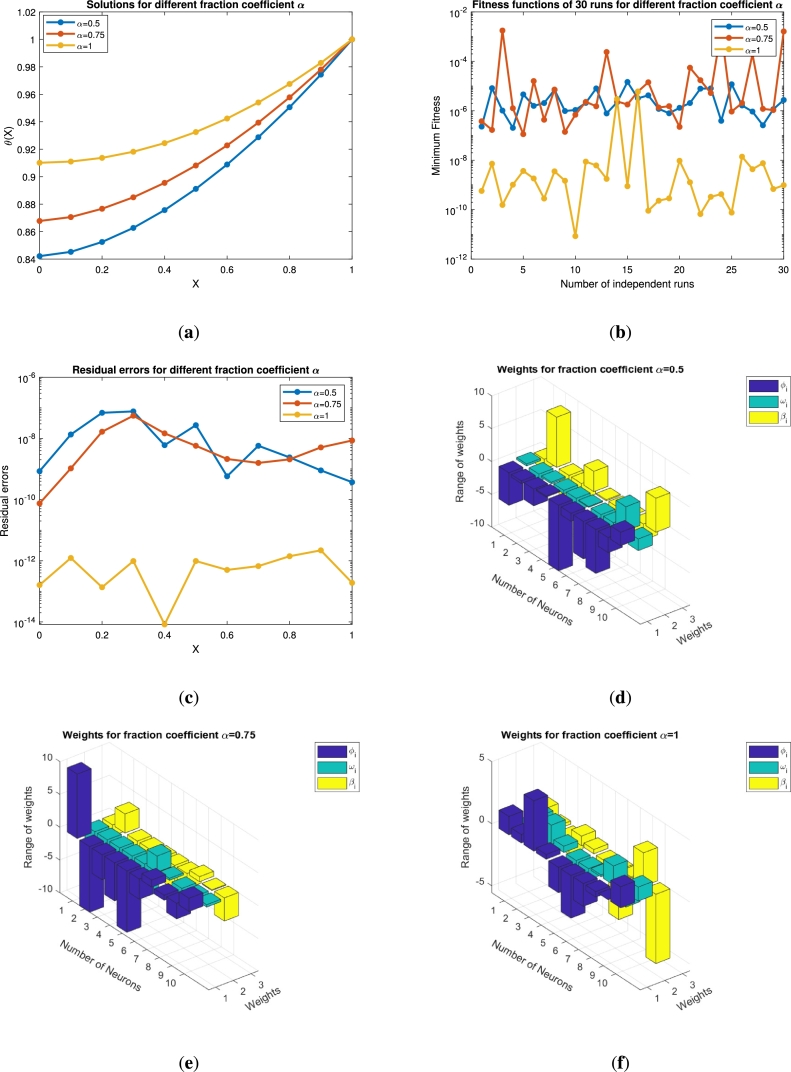
For *α* = 0.5,0.75,1 (**a**) Approximate solution of *θ*(*x*) (**b**) Fitness function (**c**) Error analysis (**d**)-(**f**) Weight functions vs neurons variation.

**Figure 8:**
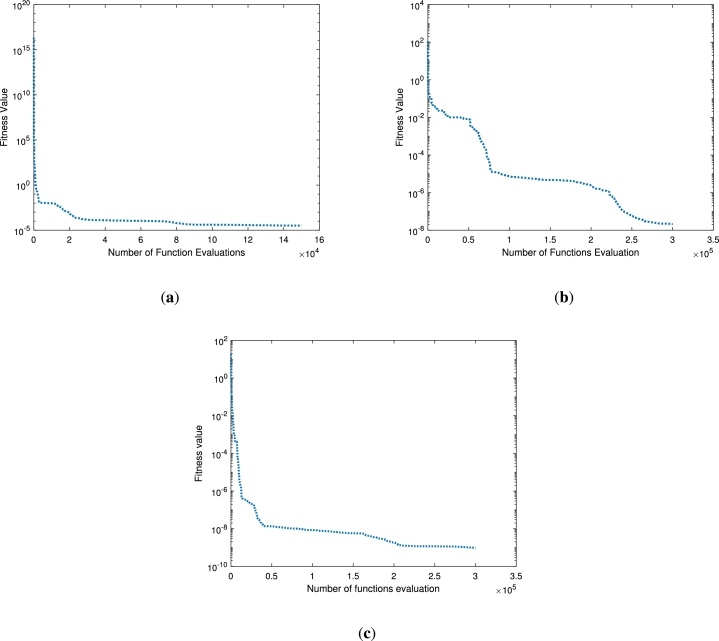
Solution convergence in each case study.

**Figure 9:**
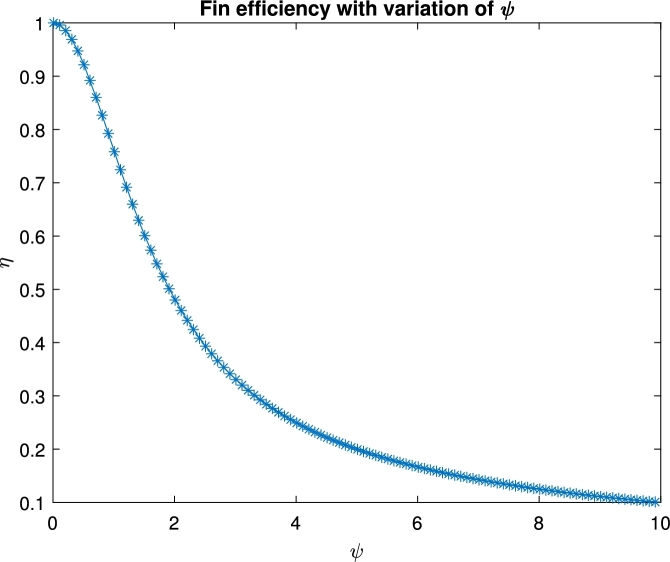
Fin efficiency.

**Figure 10:**
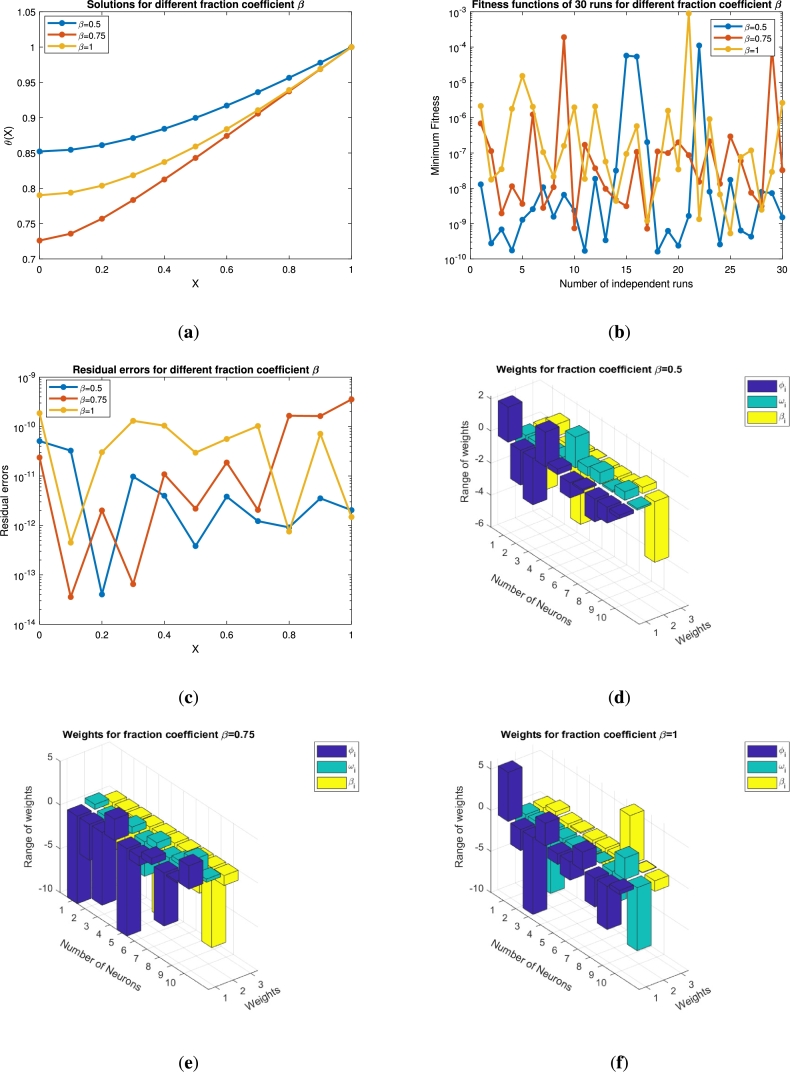
For *β* = 0.5,0.75,1 (**a**) Approximate solution of *θ*(*x*) (**b**) Fitness function (**c**) Error analysis (**d**)-(**f**) Weight functions vs neurons variation.

**Figure 11:**
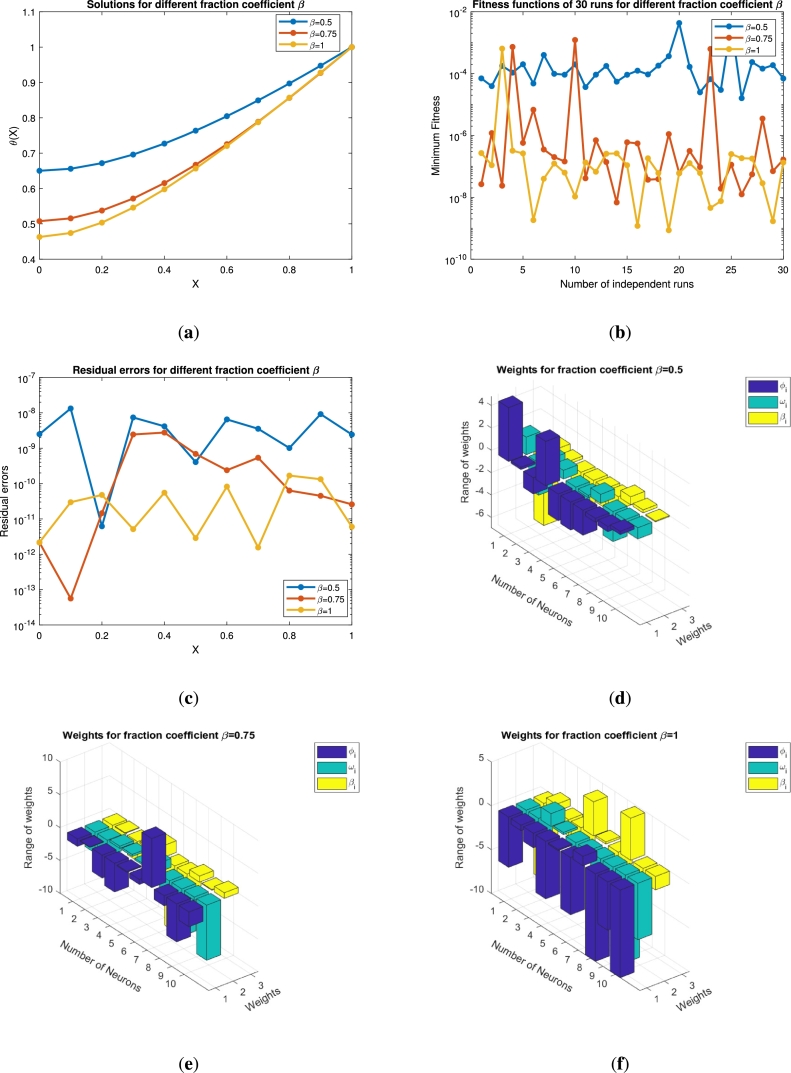
For *β* = 0.5,0.75,1 (**a**) Approximate solution of *θ*(*x*) (**b**) Fitness function (**c**) Error analysis (**d**)-(**f**) Weight functions vs neurons variation.

**Figure 12:**
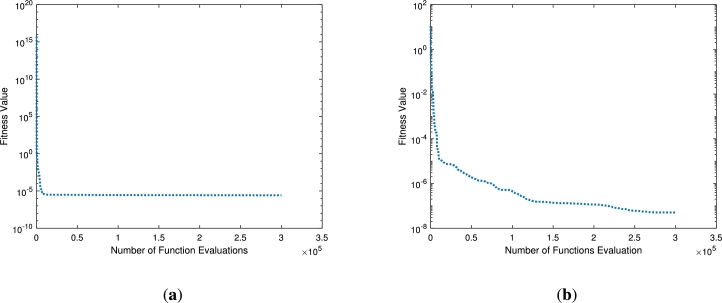
Solution convergence in each case study.

In this section the results obtained by solving the fractional order equations (14) and (25) are presented in the form of Figure 5, Figure 6, Figure 7, Figure 8, Figure 9, Figure 10, Figure 11, Figure 12 and Table 1, Table 2, Table 3, Table 4, Table 5, Table 6. The impact of various parameters on the state variables together with its residual, fitness function, weight functions and convergence analysis is presented with graphs. The efficiency of the fin is also described in this section. The numerical results for the varying values of *β*, *ψ*, and *α* together with the corresponding errors are described with tables. Finally, the results obtained are validated through Table 6 by comparing the obtained results with HPM, VIM, and LHPM.

**Table 1:** Variations in heat for various values of *β* with error analysis.

	β=0.5	*β* = 0.75	*β* = 1	*β* = 0.5	*β* = 0.75	*β* = 1
*x*	${\overset{ˆ}{\theta }}_{ann}$	${\overset{ˆ}{\theta }}_{ann}$	${\overset{ˆ}{\theta }}_{ann}$	Error	Error	Error
0	0.510425	0.573129	0.701199	5.78E-08	4.05E-07	7.41E-15
0.1	0.52186	0.583908	0.704096	7.27E-08	1.10E-07	3.24E-12
0.2	0.546378	0.605437	0.712801	2.96E-07	5.96E-08	2.56E-11
0.3	0.579537	0.633562	7.27E-01	7.07E-07	2.28E-07	8.36E-13
0.4	0.620447	0.668112	0.747788	7.63E-09	1.10E-07	3.03E-11
0.5	0.66847	0.708964	0.774205	7.41E-08	8.14E-09	5.02E-12
0.6	0.723018	0.755837	0.806696	4.97E-08	6.76E-09	1.67E-11
0.7	0.783661	0.808484	0.845378	1.39E-07	3.73E-07	3.24E-11
0.8	0.85014	0.86677	0.890386	2.60E-07	1.42E-06	4.87E-13
0.9	0.922315	0.930653	0.941872	2.02E-08	5.51E-07	7.68E-11
1	1.000114	1.000144	1	1.55E-06	2.05E-06	2.37E-11

**Table 2:** Variations in heat for various values of *ψ* with error analysis.

	ψ=0.5	*ψ* = 0.75	*ψ* = 1	*ψ* = 0.5	*ψ* = 0.75	*ψ* = 1
*x*	${\overset{ˆ}{\theta }}_{ann}$	${\overset{ˆ}{\theta }}_{ann}$	${\overset{ˆ}{\theta }}_{ann}$	Error	Error	Error
0	0.22768	0.286001	0.481415	1.69E-07	3.14E-08	2.16E-12
0.1	0.251129	0.30913	0.486152	1.05E-06	7.82E-08	2.97E-11
0.2	0.295442	0.35131	0.500421	6.92E-06	1.99E-06	4.76E-11
0.3	0.350022	0.401645	5.24E-01	4.78E-06	5.92E-06	5.13E-12
0.4	0.414407	0.460257	0.558355	2.07E-09	9.92E-07	5.52E-11
0.5	0.488591	0.527752	0.602692	2.54E-07	5.58E-07	2.87E-12
0.6	0.572248	0.60418	0.657893	1.80E-09	3.42E-09	8.21E-11
0.7	0.665118	0.689452	0.724538	3.45E-08	1.16E-09	1.55E-12
0.8	0.767191	0.783613	0.80328	6.90E-07	4.49E-08	1.68E-10
0.9	0.878686	0.886894	0.894844	7.56E-07	4.36E-08	1.33E-10
1	0.999961	0.999658	1.000001	1.30E-06	1.22E-08	5.98E-12

**Table 3:** Variations in heat for various values of *α* with error analysis.

	α=0.5	*α* = 0.75	*α* = 1	*α* = 0.5	*α* = 0.75	*α* = 1
*x*	${\overset{ˆ}{\theta }}_{ann}$	${\overset{ˆ}{\theta }}_{ann}$	${\overset{ˆ}{\theta }}_{ann}$	Error	Error	Error
0	0.84214	0.867735	0.910121	8.49E-10	7.50E-11	1.61E-13
0.1	0.845247	0.870574	0.911014	1.34E-08	1.05E-09	1.23E-12
0.2	0.85246	0.876643	0.913696	6.90E-08	1.66E-08	1.36E-13
0.3	0.862697	0.884973	9.18E-01	7.66E-08	5.54E-08	9.77E-13
0.4	0.875658	0.895494	0.924432	6.02E-09	1.47E-08	8.27E-15
0.5	0.891115	0.908136	0.932494	2.71E-08	5.76E-09	9.80E-13
0.6	0.90886	0.922791	0.942359	5.79E-10	2.12E-09	5.02E-13
0.7	0.928721	0.939361	0.954034	5.77E-09	1.58E-09	6.69E-13
0.8	0.950579	0.957777	0.967526	2.40E-09	2.07E-09	1.41E-12
0.9	0.974351	0.977999	0.982845	9.02E-10	5.10E-09	2.19E-12
1	0.999977	1.000004	1	3.71E-10	8.57E-09	1.90E-13

**Table 4:** Variations in heat for various values of *β* with error analysis.

	β=0.5	*β* = 0.75	*β* = 1	*β* = 0.5	*β* = 0.75	*β* = 1
*x*	${\overset{ˆ}{\theta }}_{ann}$	${\overset{ˆ}{\theta }}_{ann}$	${\overset{ˆ}{\theta }}_{ann}$	Error	Error	Error
0	0.852156	0.726003	0.790138	5.08E-11	2.37E-11	1.86E-10
0.1	0.854515	0.735729	0.793807	3.25E-11	3.56E-14	4.48E-13
0.2	0.86109	0.756919	0.803672	4.02E-14	2.01E-12	3.03E-11
0.3	0.871195	0.783371	8.18E-01	9.73E-12	6.48E-14	1.31E-10
0.4	0.884232	0.812473	0.837178	3.98E-12	1.08E-11	1.04E-10
0.5	0.899688	0.84297	0.859157	3.82E-13	2.18E-12	2.96E-11
0.6	0.917121	0.874178	0.883787	3.83E-12	1.87E-11	5.60E-11
0.7	0.936159	0.905693	0.910578	1.22E-12	2.06E-12	1.02E-10
0.8	0.956488	0.937263	0.939109	9.12E-13	1.65E-10	7.51E-13
0.9	0.977842	0.968731	0.96902	3.53E-12	1.63E-10	7.15E-11
1	0.999999	0.999999	1.000001	2.04E-12	3.54E-10	1.48E-12

**Table 5:** Variations in heat for various values of *β* with error analysis.

	β=0.5	*β* = 0.75	*β* = 1	*β* = 0.5	*β* = 0.75	*β* = 1
*X*	${\overset{ˆ}{\theta }}_{ann}$	${\overset{ˆ}{\theta }}_{ann}$	${\overset{ˆ}{\theta }}_{ann}$	Error	Error	Error
0	0.650042	0.507542	0.462766	2.49E-09	2.16E-12	2.16E-12
0.1	0.655802	0.515508	0.474066	1.33E-08	5.60E-14	2.97E-11
0.2	0.671725	0.537633	0.503416	6.18E-12	1.46E-11	4.76E-11
0.3	0.695976	0.571564	5.46E-01	7.41E-09	2.45E-09	5.13E-12
0.4	0.727007	0.61528	0.597664	4.17E-09	2.77E-09	5.52E-11
0.5	0.763546	0.667027	0.656452	4.05E-10	6.90E-10	2.87E-12
0.6	0.804576	0.725285	0.720195	6.53E-09	2.39E-10	8.21E-11
0.7	0.849292	0.788746	0.78741	3.55E-09	5.37E-10	1.55E-12
0.8	0.897071	0.856291	0.856975	1.01E-09	6.33E-11	1.68E-10
0.9	0.947431	0.926973	0.928046	9.21E-09	4.50E-11	1.33E-10
1	1.000002	1	1.000001	2.44E-09	2.58E-11	5.98E-12

**Table 6:** Validation of the results for *α* = 0, *ψ* = 0.2, and *β* = 1.

*ξ*	HPM [3]	VIM [9]	LHPM [46]	HCS-ANN	Error in HCS-ANN
0	0.9803	0.9803	0.980328	0.980329	2.63E-12
0.1	0.9805	0.9805	0.980524	0.980525	1.68E-11
0.2	0.9805	0.9811	0.981112	0.981113	1.39E-13
0.3	0.9821	0.982	0.982093	0.982094	9.36E-12
0.4	0.9835	0.9834	0.983467	0.983467	3.25E-12
0.5	0.9852	0.9852	0.985234	0.985234	1.32E-12
0.6	0.9874	0.9873	0.987395	0.987395	7.85E-12
0.7	0.99	0.9899	0.989951	0.989951	2.56E-12
0.8	0.9929	0.9929	0.992903	0.992903	2.50E-12
0.9	0.9963	0.9962	0.996252	0.996252	1.08E-11
1	1	1	1	1	4.56E-12

Fig. 5(a) shows the impact of the order coefficient (*β*) on the state variable *θ*. For β=0.5,0.75 and 1 the state variable shows a decreasing trend and converges towards the exact solution curve (integer order). This variation starts from θ=0.7 at x=0 and varies up to θ=1 when x⟶1. The blue line shows the results for the integer order of *β* that starts from 0.5 at x=0 and obtained its maximal value at x=1. This figure demonstrates the thermal profile of the fin at two non-integer points, where its tendency towards the convergence is proved by comparing with integer order result. Fig. 5(b) shows the minimum values for the objective function. It is clear that when x⟶1 the fitness function is at its best minimal value $10^{-11}$. This analysis demonstrates the over all performance of the objective function. The residual errors for the varying value of *β* are presented in Fig. 5(c). It is proved that the approximated and actual results are very close to each other and achieve its minimum value $10^{-14}$ at the integer order. In Figs. 5(d)-(f) the range of the weight functions is plotted for three weights $\phi_{i}$, $\omega_{i}$ and $\beta_{i}$ under the impact of the fractional coefficient β=0.5, 0.75 and 1, respectively. These figures depict the relation between the neurons in different layers. For all the values of *β* the range of weights is greater than −10 and less than 5. The number of neurons, for β=0.5 and 0.75 are nine, while for the integer order it is seven. The minimum is the weight functions smaller will be the connection between the nodes and more faster will be the rate of convergence. These weights are chosen automatically by the neural network for establishing best connection.

The impact of the thermo-geometric fin parameter *ψ* is displayed in Figs. 6(a)-6(f). The fin parameter *ψ* is chosen to be 0.5, 0.75 and 1. Fig. 6(a) shows the impact of *ψ* over the state variable *θ*. It is clear that the solution curve increases from 0.2 to 0.5 as the *ψ* values increase. The solution converges for ψ=1 and approaches the exact solution. The fitness function in Fig. 6(b) shows the minimal values up to $10^{-10}$ for ψ=1, while for ψ=0.5 and ψ=0.75 it almost has the same trend by fluctuating around $10^{-4}$. The residual errors are plotted in Fig. 6(c), where it has a convergence rate near $10^{-12}$ for ψ=1. This shows the accuracy of our implemented method. In Figs. 6(d)-(f), the weight functions, number of neurons, and their ranges are plotted. These sub-figures (d)-(f) have nine, ten, and ten neurons, respectively. The range of the neurons varies from −10 to 10. The weight functions are chosen by the neural network to minimize the fitness function and establish a best connection.

The variations in temperature with other necessary graphs are presented in Figs. 7(a)-7(f) under the impact of the dimensionless parameter of the thermal conductivity (*α*). We have chosen α=0.5,0.75,1. Physically speaking, when the thermal conductivity is enhanced, the parameter *α* also jumps, and as a result the temperature profile increases. This effect is described in Fig. 7(a). The variations move up to 0.91 when *α* approaches 1. The approximate solution at the non-integer points of *α* is 0.84 and 0.87, respectively. Similarly, the number of independent runs are plotted against the fitness functions in Fig. 7(b). It is clear that when the *α* values increase the fitness function decrease up to $10^{-12}$. These results for the state variable are justified with the residual errors in Fig. 7(c). The error plots in this sub-figure show the accuracy of our technique that varies up to $10^{-14}$, when the values of the dimensionless parameter α→1. The weight functions and the number of neurons are plotted in Figs. 7(d)-7(f). These subplots have seven, six, and ten neurons respectively, while their range varies from −10 to 10.

The over all performance for each case study is presented in Figs. 8(a)-(c). The mean square error again the number of evaluated functions are presented. In case study one as depicted in Fig. 8(a) there are $15\times 10^{4}$ functions are evaluated from the assumed bunch of functions. The minimum value for mean square error occurs at $10^{-5}$. Similarly, for the case study two and three, the minimum error as represented the fitness function occurs at $10^{-8}$ and $10^{-9}$, respectively. The evaluated functions for both the cases are $3\times 10^{5}$.

The efficiency of the fin is presented in Fig. 9. The figure depicts the fin efficiency as a function of the thermo-geometric fin parameter for distinct values of thermal conductivity. The findings of ANN match those of the precise solution for the situation of constant heat conductivity.

The solution analysis for the second problem (24) for various values of *β* is displayed in Figs. 10(a)-(f) and 11(a)-(f) by choosing $Pe=0.1,N=0.1,Nr=0.2,Q=0.2,\theta_{a}=0.1,\theta_{s}=0.3,Sp=0.2$ and $Pe=0.1,N=0.7,Nr=0.2,Q=0.2,\theta_{a}=0.1,\theta_{s}=0.1,Sp=0.2$., respectively. In both the cases we have varied the fractional order of the derivative β=0.5,0.75 and 1. When the values of *β* are increasing the solution curve converges towards the integer order solution as shown in Fig. 10(a). The fitness function and the mean square error for this case study are depicted in Figs. 10(b)-(c). Both the profiles approaching the minimum values of $10^{-10}$ and $10^{-14}$, respectively. The best values of the fitness function are depicted at β=0.5. The weight functions $\left(\phi_{i},\omega_{i},\beta_{i}\right)$ are chosen in the range of −6 to 2, −10 to 5 and −10 to 5, respectively. Similarly, the same parameter under different constant parameters as a second case is presented in Figs. 11(a)-(f). Its is clear from Fig. 11(a) that when the fractional order increases the thermal profile falls below 0.5. This interesting result for the thermal profile is achieved at β=01. The fitness function falls to $10^{-10}$ for β=1, while remains below $10^{-2}$ for other non-integer points. The residual errors for each point for the computation of the thermal profile are displayed in 11(c). The residual errors lie in the range $10^{-7}$ to $10^{-14}$. This shows the accuracy of the total performance of the implemented technique. The corresponding weights against the number of neurons are presented in Figs. 11(d)-(f). There are seven, ten and ten neurons for three different wights of β=0.5,0.75 and β=1, respectively. The fitness values against the number of functions evaluated are presented in Figs. 12(a)-(b). The minimum fitness values for the first case study is $10^{-5}$, where $3\times 10^{5}$ functions are evaluated as shown in Fig. 12(a). On the other hand the same number functions are evaluated in the second case study for a minimum value $10^{-7}$ of the fitness.

### 6.1 Tables discussion

The numerical results are presented in the form of Table 1, Table 2, Table 3, Table 4, Table 5. It is clear from Table 1 that when the values of *β* increase from fraction to integer order the temperature profile falls down towards the exact solution (that occurs at the integer order). This variation is proved in the three right-most columns of this table. The error values for integer order vary up to E−15, which is a remarkable achievement for our technique. The other two columns for β=0.5 and 0.75 are also very impressive. The thermo-geometric fin parameter *ψ* impact is numerically described in Table 2. The errors for these variations are also presented. The temperature profile falls down with larger values of *ψ* and jumps towards the exact solution. The error columns show the efficiency of our method which is E−12 at the integer order of the thermo-geometric parameter. The error shows the efficiency and accuracy of the implemented method. The impact of the thermal conductivity in terms of *α* is described in Table 3 numerically. It is clear from the first three columns that when the thermal conductivity is enhanced the corresponding temperature jumps. The accuracy of these results can be seen from the right side of the table, where the three rightmost columns are describing the error in each case. For larger values of *α* i.e. 1 the error column shows are minimal value of E−15, which very a great achievement for our method.

The approximate solution for two different cases of *β* is displayed in Table 4, Table 5. These are the approximate solutions of the second problem. It is clear that in both the cases the convergence to exact solution is much faster in the later one. It is evident that the fractional order effect directly influenced by the other constant parameters. In the later case we achieved the exact solution for all the values of *β*, while in first case these very close to the exact solution as depicted in Table 4. The error columns in both the tables touche E−14. The approximations and the corresponding errors show the accuracy of our implemented technique.

### 6.2 Section validation of results

This section is important for the technique we used in this analysis. The results we obtained through the artificial neural network probed by the hybrid Cuckoo search algorithm (HCS-ANN) are compared with the available result in Table 6. The results are described at α=0, ψ=0.2, and β=1. Three different results obtained through HPM, VIM, and Laplace HPM are compared with our results. For more accuracy, the rightmost column shows the error analysis for HCS-ANN, where our results look quite impressive up to 12th decimal place. At ξ=0, we see that the HCS-ANN result is more accurate as compared to the other techniques. This variation can be observed in other values as ξ⟶1.

## 7 Conclusions

We analyzed the fractional order straight convective fin with HCS. The outcome is presented in the form of figures and tables. The impact of the parameters *α*, *β*, and *ψ* are explained with graphs and tables for 100 runs. The fin efficiency is also described for *ψ*. Based on our analysis, we recommend the following.

• The increasing values of *β* cause to reduce the thermal profile.
• The efficiency of the fin is higher at the lower values of *ψ*.
• With less computational effort by choosing a single hidden layer a high level of accuracy up to E−14 is observed.
• The performance in each case study proves the HCS convergence.
• The HCS results are validated in the last table by comparing with the available literature.
• The error vary up to E−15 for the integer order of the fractional coefficient *β*.
• For nonlinear problems of fractional order arsing in engineering and related applied sciences, we recommend HCS.

## Abbreviations

Following are the abbreviations used in this work:

**Table:** 

*Nr*	radiation parameter	*A*_*c*_	Cross sectional area of the fin (m^2^)
*k*	Thermal conductivity	*b*	length of the fin (m)
*h*	Convective heat coefficient	*P*	perimeter of the fin (m)
*Da*	Darcy number	*K*(*T*)	Temperature dependent thermal conductivity
*Sp*	Porosity parameter	*λ*	Variation in thermal conductivity
*T*_*a*_	Ambient temperature (K)	*T*_*s*_	Surface temperature (K)
*ξ*	Independent variable (dimensionless)	*x*	Distance (m)
*C*_*p*_	Specific heat coefficient	*θ*_*b*_	Base temperature (K)
*ϵ*	Surface emissivity	*y*(*x*)	Analytic function
*θ*	Dimensionless temperature	$\overset{ˆ}{\theta }$	Temperature approximation
*β*	Fractional parameter	*ψ*	Thermo-geometric parameter
*ϕ*_*i*_,*ω*_*i*_,*β*_*i*_	Weight functions	*η*	Fin efficiency
*H*(*u*)	Heaviside function	*Pe*	Peclet number
*N*	Condition-convection parameter	*m*	Number of neurons
*f*(*t*)	Sigmoid function	E	Fitness function
*s*	Step size	*ϵ*_1_	Scaling factor
*t*	Time (s)	*α*_1_	Scaling factor
⊗	Element-wise product	*μ*	Emigration rate
*p*_*a*_	Probability of egg discovery	*T*	Variable temperature (K)

## CRediT authorship contribution statement

**Waseem:** Conceptualization, Formal analysis, Investigation, Project administration, Resources, Supervision, Validation, Writing – original draft. **Asad Ullah:** Conceptualization, Formal analysis, Investigation, Methodology, Project administration, Software, Validation, Visualization, Writing – original draft, Writing – review & editing. **Sabir Ali:** Data curation, Formal analysis, Software, Validation, Visualization, Writing – review & editing. **Fuad A. Awwad:** Conceptualization, Data curation, Formal analysis, Funding acquisition, Software, Validation, Visualization, Writing – review & editing. **Emad A.A. Ismail:** Conceptualization, Formal analysis, Funding acquisition, Software, Visualization, Writing – review & editing.

## Declaration of Competing Interest

The authors declare that they have no known competing financial interests or personal relationships that could have appeared to influence the work reported in this paper.

## Data Availability

No data is used in this study.
